# Nutritional strategies during gastrointestinal dysfunction

**DOI:** 10.1097/MCC.0000000000001052

**Published:** 2023-06-14

**Authors:** Rob J.J. van Gassel, Julia L.M. Bels, Marcel C.G. van de Poll

**Affiliations:** a Department of Intensive Care Medicine; b Department of Surgery, Maastricht University Medical Centre +; c NUTRIM School for Nutrition and Translational Research in Metabolism, Maastricht University, The Netherlands

**Keywords:** critical illness, gastrointestinal dysfunction, malabsorption, nutrition

## Abstract

Gastrointestinal (GI) dysfunction is common among critically ill patients and is associated with poor outcomes. In particular, nutrient delivery can be impaired in patients with GI dysfunction and pose a significant challenge to clinicians in daily clinical practice. This review aims to summarize the impact of GI dysfunction on nutrition therapy during critical illness and provide an update on recent advances in nutritional strategies during gastrointestinal dysfunction.

**Recent findings:**

Although prognostic gastrointestinal dysfunction scoring systems exist, a lack of clear, uniform definitions of GI dysfunction limits diagnosis and subsequent adequate treatment. Recent studies have further investigated separate components of GI dysfunction in ICU patients, including the role of altered GI motility, nutrient digestion and absorption and the metabolic consequences of gut dysfunction. Various strategies to improve nutrient delivery are discussed. However, the evidence supporting their routine use is sometimes lacking.

**Summary:**

GI dysfunction frequently occurs during critical illness and negatively affects nutrition therapy. Strategies to improve nutrient delivery during GI dysfunction are available, though more research into the diagnosis and pathophysiology of GI dysfunction will likely further improve patient outcomes.

## INTRODUCTION

In critically ill patients admitted to an intensive care unit, nutritional support is part of routine care [1,2]. Nutrition support aims to meet the metabolic demands of patients during their disease state to maintain normal physiology and prevent the body from depleting its metabolic reserves, resulting in severe skeletal muscle wasting and loss of functional capacity in patients. The potential impact of nutrition therapy on the long-term functional outcomes of patients has gained increasing interest among clinicians, researchers, and patients. Nevertheless, much is still unknown about nutritional support's optimal dosage, timing, and composition during critical illness [3^▪^]. One complicating factor that can present a significant challenge in providing adequate nutritional support to critically ill patients is the occurrence of gastrointestinal (GI) dysfunction. GI dysfunction is common during critical illness and can significantly impact the provision and subsequent uptake and handling of enterally provided nutrients and patient outcomes [4]. In the current review, we will address the impact of GI dysfunction during critical illness on nutrition support and discuss potential nutritional strategies during GI dysfunction. 

**Box 1:**
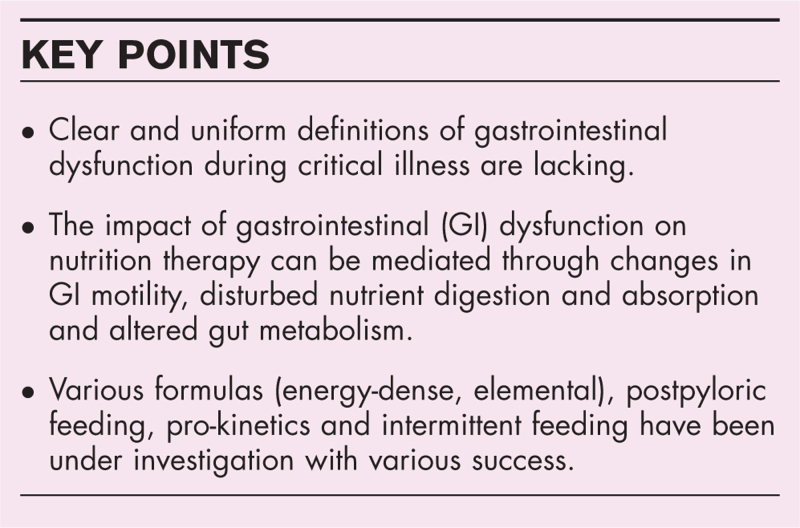
no caption available

## DEFINING GASTROINTESTINAL DYSFUNCTION IN CRITICAL CARE

Although gastrointestinal dysfunction is common among critically ill patients, the exact incidence of gastrointestinal dysfunction in critical care remains unknown. This is largely due to the lack of clear definitions and accurate diagnostic tools to assess GI dysfunction. In order to provide better tools to define GI dysfunction in routine clinical practice, the acute gastrointestinal injury (AGI) score was proposed in 2012 [5]. In a recent study, Reintam Blaser *et al.*[6^▪▪^] updated the AGI score and developed the gastrointestinal dysfunction score (GIDS). Clinically available abdominal symptoms, including gastric residual volume, diarrhea, ileus symptoms, GI bleeding, and intra-abdominal pressure, are used in a scoring system ranging from grade 0 to 4. Whilst this scoring system can predict mortality in critically ill patients, it only focuses on GI dysfunction symptoms routinely measured in clinical practice and mostly related to disturbed motility. However, the pathophysiology and subsequent manifestations of ICU-related GI dysfunction can vary strongly, resulting not only from changes in GI motility but also impaired digestion and absorption of enteral nutrition, an altered postprandial entero-endocrine response, and changes in gut mucosal integrity, mesenteric perfusion, and intra-abdominal pressure [7]. This great heterogeneity makes it difficult to define, recognize, and subsequently adjust the treatment for critically ill patients with GI dysfunction [8]. Although studies have investigated the potential role of biomarkers such as I-FAB (intestinal fatty acid binding protein), which is expressed in the gut epithelial layer and released during injury, and citrulline, which is converted from glutamine by the gut enterocyte, many biomarkers are proven to be limited in their ability to accurately identify patients with GI dysfunction and have not found their way into routine clinical practice [6^▪▪^,^9^]. Therefore, there remains a strong need for accurate biomarkers or tools to identify the various forms of GI dysfunction.

## GASTROINTESTINAL DYSFUNCTION AND CLINICAL OUTCOMES

The link between GI dysfunction, especially when multiple abdominal symptoms occur concomitantly, and increased mortality and morbidity has been described before [10]. It remains unclear whether GI dysfunction is the cause of poorer outcomes in critically ill patients or is simply a marker for disease severity. One explanation of why GI dysfunction might negatively impact patient outcomes is that it might limit the ability to provide nutrition therapy to patients adequately and that adequate nutrient uptake and handling might be disturbed in patients with severe GI dysfunction. In a prospective observational study, ICU patients with higher acute gastrointestinal injury (AGI) grades early in the ICU disease course, indicating increasing severity of GI dysfunction, also received fewer calories [11^▪^]. Furthermore, higher AGI grade and lower caloric intake were associated with increased 28-day mortality. Other mostly observational studies have demonstrated an association between inadequate caloric and protein intake during ICU stay with increased mortality and morbidity in patients [12,13]. Therefore, if GI dysfunction could affect patient outcomes through poor nutrient handling, targeted nutritional interventions that help alleviate or overcome GI dysfunction may improve critical illness admission clinical outcomes. Developing such potentially impactful interventions requires a better understanding of the underlying mechanisms and interactions between GI dysfunction and nutrient handling.

## GASTROINTESTINAL DYSFUNCTION AND NUTRIENT HANDLING

Gastrointestinal dysfunction can negatively impact the provision and uptake of enteral nutrition, leading to malnutrition and poor outcomes in critically ill patients. Here, we discuss the main mechanisms through which GI dysfunction can lead to malnutrition.

### Disturbed gastrointestinal motility

Nutritional support during critical illness is tailored to the individual patients by setting nutritional targets based on either calculated or measured caloric and protein demands [1,2]. In daily clinical practice, however, the amount of nutrition provided often falls below the amount prescribed [14]. This gap between the amount of nutrition prescribed and the amount provided is termed nutritional adequacy, and disturbed gastrointestinal motility can be one of the underlying causes of poor nutritional adequacy. The occurrence of delayed gastric emptying and high gastric residual volumes (GRV) is a frequent cause of enteral nutrition interruptions [15]. In an observational study, high GRV was the second most common cause of enteral nutrition interruption (17% of EN interruptions), followed by the occurrence of diarrhea (10% of EN interruptions) [16]. Consequently, these patients suffered significant caloric and protein deficits compared to patients without these symptoms. A recent posthoc analysis of The Augmented vs. Routine approach to Giving Energy Trial (TARGET) investigated the incidence of GI dysfunction during enteral nutrition [17^▪▪^]. In this study, GI dysfunction was defined as GRVs of more than 250 ml and occurred in almost half of all patients (46%), and occurred more frequently in males, younger patients, and the more severely ill. In contrast to observations made in other studies, there was no difference in the amount of calories delivered between patients with and without GI dysfunction. Although disturbed GI motility can lead to poor nutritional adequacy, it can be recognized relatively easily, and different strategies are available to improve nutritional care for these patients. These interventions are discussed later.

### Maldigestion and malabsorption

Aside from disturbed gastrointestinal motility, GI dysfunction can also occur at nutrient digestion and absorption levels. Earlier studies using isotopically labeled molecules to assess enteral nutrition uptake have demonstrated that compared to healthy controls, the uptake of dietary glucose, lipids, and protein is impaired in critically ill patients [18–20]. Opposed to disturbed GI motility, maldigestion and malabsorption often occur occult, as readily available tests to assess nutrient uptake are lacking. Furthermore, physiological studies in critically ill patients demonstrate great variation in the uptake of dietary amino acids, for instance, varying greatly between patients and over time [21]. Moreover, different nutrient classes can be affected separately, as impaired uptake of dietary amino acids does not necessarily coincide with impaired glucose uptake [22^▪^]. More recently, there has been increasing interest in the uptake and metabolism of dietary protein. Dietary protein has gained interest because of its potential to attenuate skeletal muscle wasting during critical illness and improve functional recovery of patients, though robust prospective evidence on this is still lacking [23]. Chapple *et al.*[24^▪▪^] recently investigated protein digestion and absorption rates and the subsequent contribution of dietary protein to skeletal muscle protein synthesis in critical illness. Using intrinsically labeled protein administered through a postpyloric feeding tube, they could assess in vivo dietary protein digestion and absorption rates in both healthy subjects and critically ill patients. Approximately half of the dietary protein provided appeared in the systemic circulation following protein digestion and amino acid absorption and was not significantly different between the critically ill patients and healthy controls. Although protein absorption rates were similar, postprandial increase in muscle protein synthesis was significantly blunted in critically ill patients. These data suggest that protein malabsorption might not be as severe during critical illness as previously thought, but it also raises the question of whether higher doses of dietary protein are beneficial to overcome the blunted response of muscle protein synthesis [25,26].

### Metabolic consequences of gastrointestinal dysfunction

Aside from motility, digestion, and absorption of nutrients, the gastrointestinal tract is also active as a metabolic and endocrine organ concerning postprandial nutrient handling. Although much is still unknown, there is increasing insight into the potential metabolic complications of GI dysfunction [7]. Examples of these include the observation that the occurrence of diarrhea is associated with the occurrence of cholestasis, which might be mediated through a disturbed gut–liver axis [27]. Bile acids are considered the potential mediators of this disturbed gut–liver axis. Bile acids are produced in the liver and released into the gut following gallbladder contraction. During critical illness, gallbladder contraction is diminished and associated with increased plasma bile acid levels and reduced levels of postprandial fibroblast growth factor 19 (FGF19), a gut hormone normally responsible for the suppression of bile acid synthesis in the liver to prevent toxic levels of bile acids in the liver [28]. The loss of FGF19 as a feedback loop mechanism suppressing bile acid synthesis in the liver might explain the elevated plasma bile acid levels observed during critical illness, which in turn have been associated with worse outcomes [29]. GI dysfunction can also impact the gut microbiome, which undergoes various changes in critically ill patients. While there are associations between the altered gut microbiome and poor outcomes, including more frequent infectious complications or even death, this is an emerging field of research for which the clinical impact is still unclear [30]. Alterations in the gut endocrine system could also be considered a form of GI dysfunction. An example is glucagonlike peptide-1 (GLP-1), a gut-derived hormone that stimulates insulin secretion, which is elevated in critically ill patients and a predictor of death and poor long-term functional outcomes [31]. These examples illustrate that the metabolic consequences of GI dysfunction go beyond nutrient provision and uptake. Further exploration into the underlying pathophysiological mechanisms might help develop novel (nutritional) strategies to restore the gut-liver cross-talk, microbiome, or gut-derived endocrine response in ICU patients.

## NUTRITIONAL STRATEGIES DURING GASTROINTESTINAL DYSFUNCTION

**FIGURE 1:**
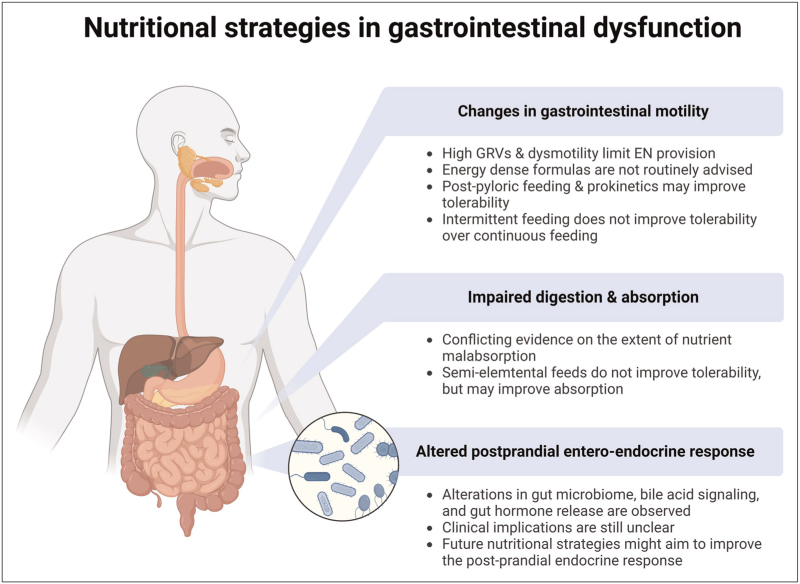
Summary of nutritional strategies in gastrointestinal dysfunction.

To mitigate the impact of gastrointestinal dysfunction, various nutritional strategies exist to help minimize its impact on nutrition delivery and patients’ health (Fig. 1). We will briefly discuss the rationale and evidence to support these strategies in routine practice. Energy-dense formulas containing a higher caloric content per ml of enteral nutrition have been proposed in patients who can tolerate lower volumes of enteral nutrition due to GI dysfunction. In the TARGET trial comparing normal (1.0 kcal/ml) vs. energy-dense (1.5 kcal/ml) nutrition in a general ICU population, energy-dense nutrition did not result in improved survival for critically ill patients [32]. In a posthoc analysis comparing patients with or without GI dysfunction, energy-dense formulas did not improve caloric delivery compared to the standard energy formula [17^▪▪^]. A recent meta-analysis demonstrated that higher energy delivery was associated with a significant increase in the incidence of GI dysfunction and large GRVs [33]. This has been corroborated in a smaller physiological study, which demonstrated that energy-dense formulas could result in more gastric retention without improving energy delivery or glucose absorption [34^▪^]. Based on these data, it can be concluded that using energy-dense formulas should be considered carefully, especially in critically ill patients with GI dysfunction. Postpyloric feeding, which bypasses the stomach, is another strategy for patients with GI dysfunction, especially in the case of gastroparesis with increased GRVs. In a retrospective observational study in major burns patients, feeding via a postpyloric feeding tube resulted in higher nutritional adequacy when compared to gastric feeding for both energy and protein delivery [35]. An observational study comparing nutritional practices in pediatric intensive care units showed that patients with a postpyloric feeding tube achieved higher enteral intake when compared to patients with an intragastric feeding tube [36]. Although intragastric feeding is often the most simple and preferred route for enteral nutrition in mechanically ventilated patients, postpyloric feeding can be considered in patients with GI dysfunction, particularly in patients with gastroparesis [37]. Prokinetics aimed at improving gastrointestinal motility are another option to improve nutrient delivery in critically ill patients. In the aforementioned posthoc analysis of the TARGET trial, the administration of prokinetics to patients with GI dysfunction was even associated with improved survival [17^▪▪^]. Another strategy that has been proposed to overcome nutrition malabsorption during GI dysfunction is the use of elemental feeds. Elemental feeds are nutritional formulas containing oligopeptides or medium-chain triglycerides rather than their polymeric counterparts and are assumed to be more readily absorbed by the gut. In a recent randomized controlled trial, brain-injury patients were randomized between an elemental or polymeric formula [38]. The use of elemental feeds did not result in more energy intake, nor did it result in less GI dysfunction in terms of gastroparesis or diarrhea, in line with a previous study in a general ICU population [39]. Actual absorption of the elemental vs. polymeric formulas was not assessed and remained an open question. The absorption kinetics of single amino acids versus intact protein was recently assessed using stable isotope methodology in healthy subjects [40]. Ingestion of free amino acids rather than intact protein resulted in a significantly higher systemic release of diet-derived amino acids into the circulation and a stronger net anabolic effect on whole-body but not skeletal muscle protein metabolism. Whether the improved absorption kinetics of these elemental feeds could benefit critically ill patients remains to be studied. Finally, intermittent vs. continuous feeding has been under investigation recently, as intermittent feeding might resemble nutrition delivery under healthy circumstances better and stimulate the normal physiological postprandial responses. However, intermittent feeding had similar incidences of gastric intolerance compared to continuous feeding and did not result in increased nutrition delivery of plasma amino acids [41,42].

## CONCLUSION

Gastrointestinal dysfunction is common among critically ill patients and can negatively impact nutrition delivery, uptake, and handling. This should be accounted for when providing nutrition support to critically ill patients with GI dysfunction, but the heterogeneity in manifestations and lack of clear definitions and accurate and routinely available tests represent a significant challenge for ICU clinicians. Some strategies are available to overcome GI dysfunction, but further research into new diagnostic and therapeutic strategies and their impact on clinical and patient-relevant outcomes is needed.

## Acknowledgements

*None.*

### Financial support and sponsorship

*None.*

### Conflicts of interest

*There are no conflicts of interest.*

## REFERENCES AND RECOMMENDED READING

Papers of particular interest, published within the annual period of review, have been highlighted as:

▪ of special interest
▪▪ of outstanding interest
